# Enhanced electrocardiogram classification using Gramian angular field transformation with multi-lead analysis and segmentation techniques

**DOI:** 10.1016/j.mex.2025.103297

**Published:** 2025-04-08

**Authors:** Gi-Won Yoon, Segyeong Joo

**Affiliations:** Department of Biomedical Engineering, Asan Medical Institute of Convergence Science and Technology, Asan Medical Center, University of Ulsan College of Medicine, Seoul, Republic of Korea

**Keywords:** Electrocardiogram, Atrial fibrillation, Gramian angular field, Classification, Deep Learning, EGAFCovNext

## Abstract

Conventional manual or feature-based ECG analysis methods are limited by time inefficiencies and human error. This study explores the potential of transforming 1D signals into 2D Gramian Angular Field (GAF) images for improved classification of four ECG categories: Atrial Fibrillation (AFib), Left Ventricular Hypertrophy (LVH), Right Ventricular Hypertrophy (RVH), and Normal ECG.

• The study employed GAF transformations to convert 1D ECG signals into 2D representations at three resolutions: 5000 × 5000, 512 × 512, and 256 × 256 pixels.
• Segmentation methods were applied to enhance feature localization.
• The ConvNext deep learning model, optimized for image classification, was used to evaluate the transformed ECG images, with performance assessed through accuracy, precision, recall, and F1-score metrics.

The 512 × 512 resolution achieved the optimal balance between computational efficiency and accuracy. F1-score for AFib, LVH, RVH and Normal ECG were 0.781, 0.71, 0.521 and 0.792 respectively. Segmentation methods improved classification performance, especially in detecting conditions like LVH and RVH. The 5000 × 5000 resolution offered the highest accuracy but was computationally intensive, whereas the 256 × 256 resolution showed reduced accuracy due to loss details.

## Graphical abstract

**Figure:**
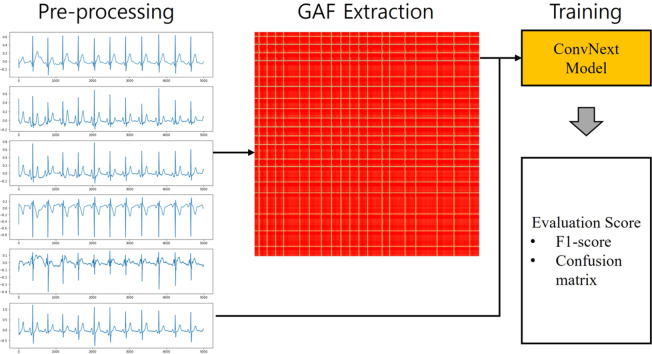

Specifications table

**Table:** 

Subject area:	Bioinformatics
More specific subject area:	Electrocardiogram
Name of your method:	EGAFCovNext
Name and reference of original method:	
Resource availability:	Hardware: 4090 super GPU Data: https://physionet.org/content/ptb-xl/1.0.3/ Software: Tensorflow and python

## Background

Cardiovascular disease (CVD) encompasses a broad range of conditions affecting the heart and blood vessels, including coronary artery disease, heart failure, arrhythmias, and hypertensive heart disease [1]. CVD remains the leading cause of death globally, responsible for approximately 17.9 million deaths annually, according to the World Health Organization [2]. Early detection and accurate diagnosis of CVD are crucial for effective treatment and reducing the risk of severe outcomes such as heart attacks [3,4]. Electrocardiogram (ECG) signals are a primary diagnostic tool for various cardiovascular conditions [5].

To overcome these limitations, modern ECG classification methods have increasingly leveraged machine learning and deep learning techniques [[6], [7], [8], [9]]. Carrilo-Alarcon et al. addresses the challenge of classifying arrhythmias from unbalanced ECG data using metaheuristic optimization techniques. The authors propose a novel approach that optimizes model parameters to enhance classification performance on unbalanced datasets [10]. These approaches aim to automate the interpretation of ECG signals, improving both the speed and accuracy of diagnosis. Early machine learning methods typically involved feature extraction, where specific characteristics of the ECG signal—such as heart rate, and intervals—were manually extracted and used as inputs for classifiers like Support Vector Machines (SVM) or Random Forests [11].

In recent years, deep learning has emerged as a powerful tool for ECG classification, capable of automatically learning features from raw data without the need for manual feature extraction. Convolutional Neural Networks (CNNs), in particular, have shown great promise in this domain. Xuan Hua et al. presented a novel approach to ECG classification using a one-dimensional convolutional neural network (1D-CNN) to accurately detect arrhythmias. The method focuses on directly processing 1D ECG signals without transforming them into 2D representations. Where this research highlights the potential of end-to-end 1D deep learning approaches for ECG analysis [12]. CNNs have been successfully applied to various ECG classification tasks, including the detection of arrhythmias, myocardial infarction, and other cardiac conditions [[13], [14], [15]]. Building on the foundation of CNNs, more complex models like ResNet and ConvNext have been developed [16,17].

Despite these advancements, the representation of ECG signals remains a critical challenge. The 1D nature of ECG signals limits the application of advanced image-based techniques that have revolutionized fields like computer vision [18,19]. To address this, this study explores the use of Gramian Angular Field (GAF) transformation, which converts raw 1D ECG signals into 2D images. This transformation preserves the temporal dynamics of the ECG signal while enabling the use of image-based deep learning models for classification. By transforming ECG data into a 2D format, we can leverage the strengths of CNNs and other deep learning architectures designed for image processing.

For related works, there have been attempts to utilize Gramian Angular Field (GAF) transformation in the context of ECG classification [20,21]. In these studies, GAF has been used to convert ECG signals into 2D representations, enabling the application of image-based machine learning techniques. However, a significant limitation of these approaches is that the GAF transformation was often applied on a beat-wise basis [22]. This means that the GAF features were calculated for individual heartbeats, without capturing the temporal relationships between consecutive beats. Such an approach is restrictive, particularly for cardiovascular conditions like Left Ventricular Hypertrophy (LVH) and Right Ventricular Hypertrophy (RVH), where the interaction between beats is crucial for accurate diagnosis [23]. Additionally, most of these studies calculated GAF on a single ECG lead rather than using lead-to-lead transformations, which further limits the ability to capture the full complexity of the heart's electrical activity across different perspectives.

## Method details

**Fig. 1:**
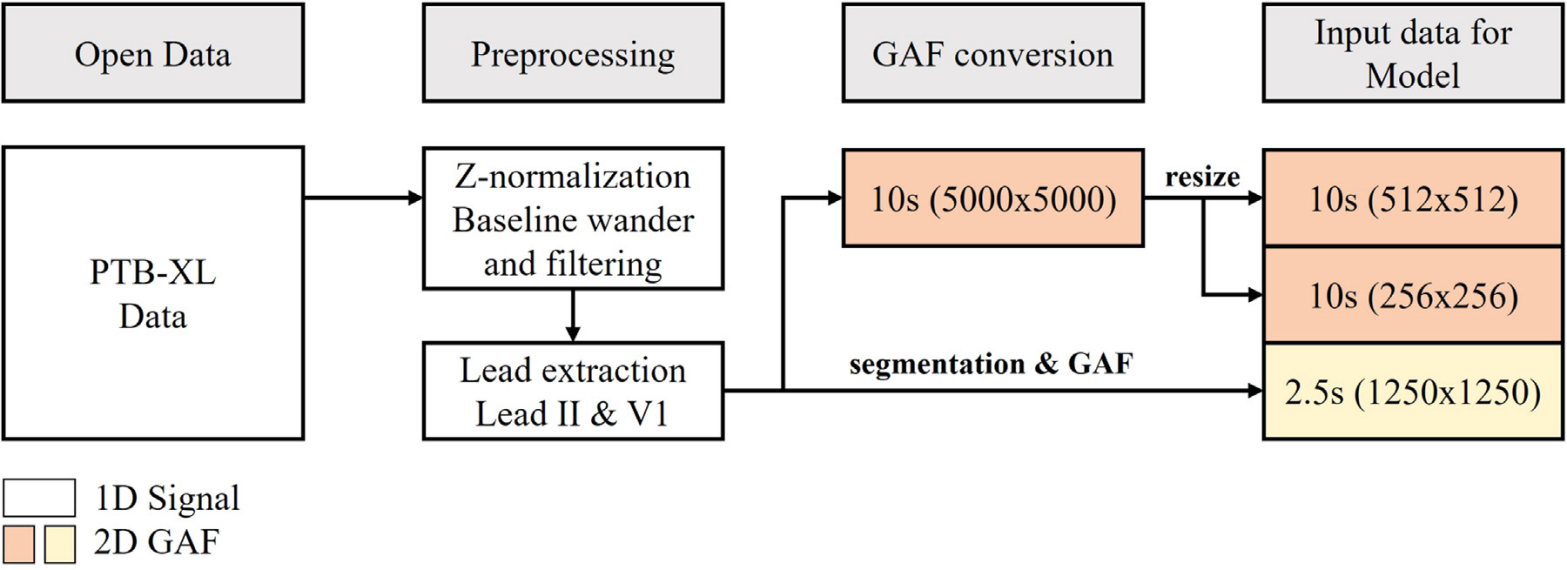
Overview of the data preparation.

The experimental workflow for this study consists of several key stages, starting from data acquisition to the final evaluation of classification performance. The process begins with the use of the PTB-XL dataset, a comprehensive open-source ECG database that provides multi-lead ECG recordings. The data undergoes preprocessing, including filtering and normalization steps to prepare the signals for further analysis (Fig. 1).

**Fig. 2:**
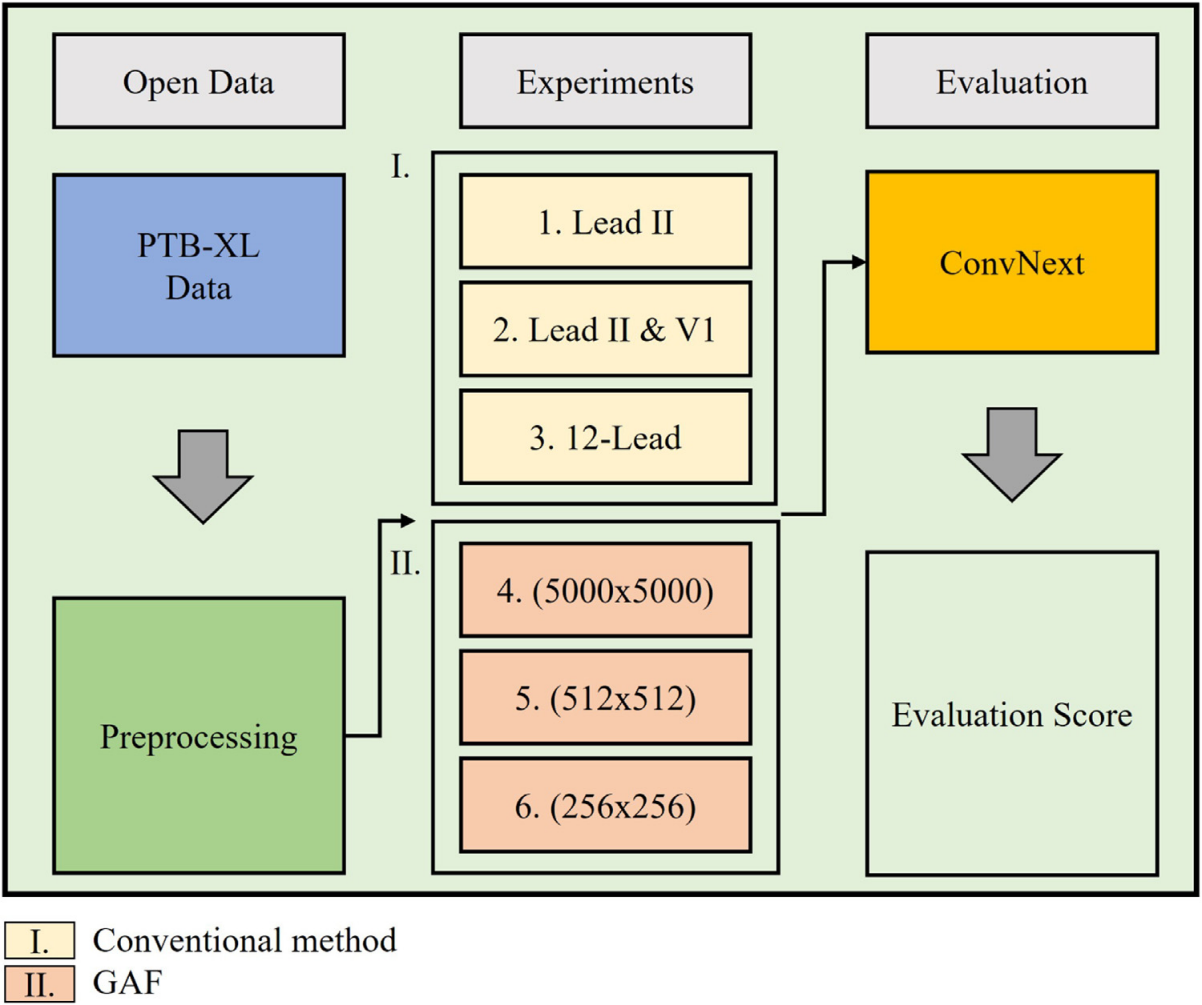
Overview of the experiment.

In the experimental setup, three configurations of ECG leads are considered: single Lead II, dual leads (Lead II and V1), and the full 12-lead ECG, to assess the impact of lead selection on classification performance. The ECG signals are transformed into Gramian Angular Fields, converting the 1D time-series data into 2D image representations. Three different GAF sizes—5000 × 5000, 512 × 512, and 256 × 256—are evaluated to determine the optimal image resolution for model performance, balancing between computational efficiency and accuracy. The segmentation process involves dividing the raw ECG signal into smaller segments of 1250 sampling points each, which corresponds to 2.5 s of signal duration at a 500 Hz sampling rate (Fig. 2).

Finally, the classification performance is evaluated using metrics such as accuracy, precision, recall, and F1-score to assess the effectiveness of the different lead configurations and GAF sizes. The results guide the selection of the most suitable approach for ECG classification, highlighting the potential of using multi-lead GAF transformations combined with advanced deep learning models for diagnosing cardiovascular conditions.

### Data collection

**Fig. 3:**
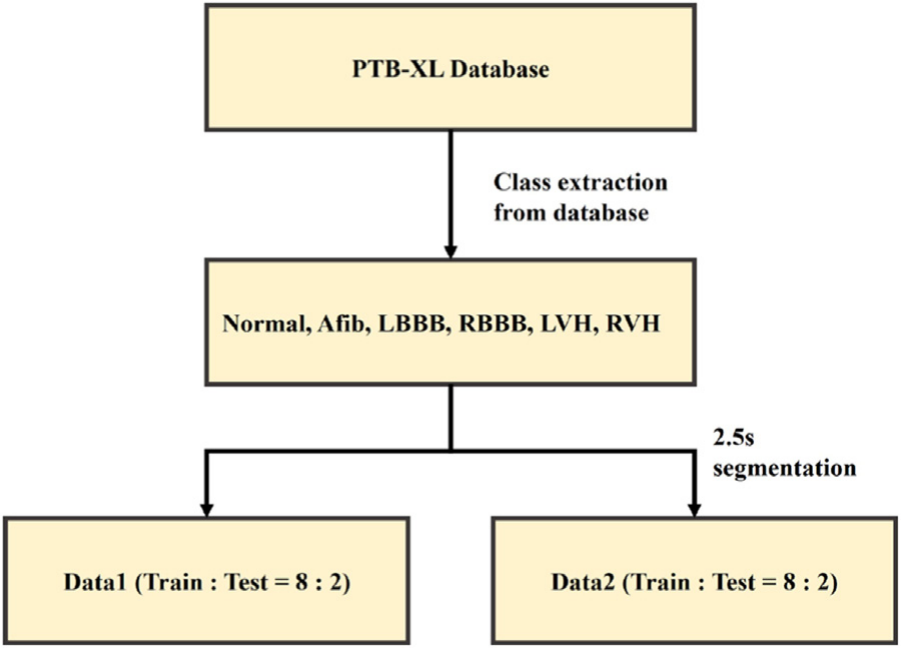
Dataset split process. Normal, Afib, RVH, LVH training set was split into a validation set at a ratio of 8:2.

ECG data corresponding to the four classes (AFib, LVH, RVH, and Normal) were collected from PTB-XL dataset [24]. The 12-lead ECG data used in this study are the PTB-XL dataset, which were publicly available and provided by the PhysioNet [24]. Table 1 lists the clinical and demographic features of each datasets. The PTB-XL dataset contains 21,837 records obtained from 18,885 patients. The duration of records in the PTB-XL dataset are 10 s. The sampling rate of 500 Hz, and the sampling points were 5000 in each record. PTB-XL dataset has 7528 normal ECG records, 1514 records of AFib, 2137 records of LVH and 126 records of RVH. As shown in Fig. 3. The training set was split into a validation set at a ratio of 8:2. The dataset was divided into training, validation, and test sets to ensure robust evaluation of the classification models.

**Table 1:** Demographic description of PTB-XL dataset.

Clinical Characteristics	PTB-XL database	Description
Age (years)	59.83 ± 16.95	
Gender (male: female)	11,379 : 10,458	
Weight (Kg)	70.99 ± 15.97	
Height (cm)	166.71 ± 10.86	

**Diagnostic statement**		

NORM	9528	Normal ECG
STTC	5788	ST/T-Change
HYP	2819	Hypertrophy
MI	6886	Myocardial Infarcation
CD	5772	Conduction Disturbance

**Rhythm statement**		

SR	16,782	Sinus Rhythm
AFIB	1514	Atrial Fibrillation
ST	826	Sinus Tachycardia
SA	772	Sinus Arrhythmia
SB	637	Sinus Bradycardia
PACE	296	Normal Functioning Artificial Pacemaker
SVA	157	Supraventricular Arrhythmia
BIGU	82	Bigeminal Pattern
AF	73	Atrial Flutter
SVT	27	Supraventricular Tachycardia
PSVT	24	Paroxysmal Supraventricular Tachycardia
TRIGU	20	Trigeminal Pattern

### Gramian angular field transformation

The raw 1D ECG signals were transformed into 2D images using the Gramian Angular Field (GAF) technique. Gramian Angular Field (GAF) is a technique for encoding time-series data, such as ECG signals, into a 2D matrix that captures temporal dependencies and relationships between different time points. The GAF represents the original time-series data in polar coordinates and leverages trigonometric functions to construct a new image representation. There are two main types of GAF: Gramian Angular Summation Field (GASF) and Gramian Angular Difference Field (GADF). GASF utilizes the cosine function to calculate the pairwise angular summation between each point in the time series.

Mathematically, it is defined as

(1) $$GASF=cos\left(\phi_{i}+\phi_{j}\right)$$

where $\phi_{i}$ and $\phi_{j}$ are the angles corresponding to the normalized values of the time-series data points. This form of GAF captures the magnitude and orientation of data points, emphasizing the similarity and accumulation patterns between them. Conversely, GADF employs the sine function to capture the angular differences between data points, defined as

(2) $$GADF=sin\left(\phi_{i}-\phi_{j}\right)$$

This difference-based approach provides a focus on the contrast and change dynamics between time points, which can be crucial for identifying anomalies or shifts in ECG patterns. By converting 1D time-series data into 2D representations, GAF allows for the application of image-based deep learning models, making it a powerful tool for tasks like ECG classification, where complex temporal relationships and patterns need to be understood and analyzed. In our study, we used two different leads to calculate GAFs instead of one. This approach was taken to capture the changes in time points between the leads.

#### GAF size variations and segmentation

Three different sizes of GAF images were generated for experimentation:

5000 × 5000: The original size, preserving the highest level of detail.

512 × 512: A reduced size, balancing detail preservation and computational efficiency.

256 × 256: A further reduced size, offering potential computational advantages with minimal loss of detail.

**Fig. 4:**
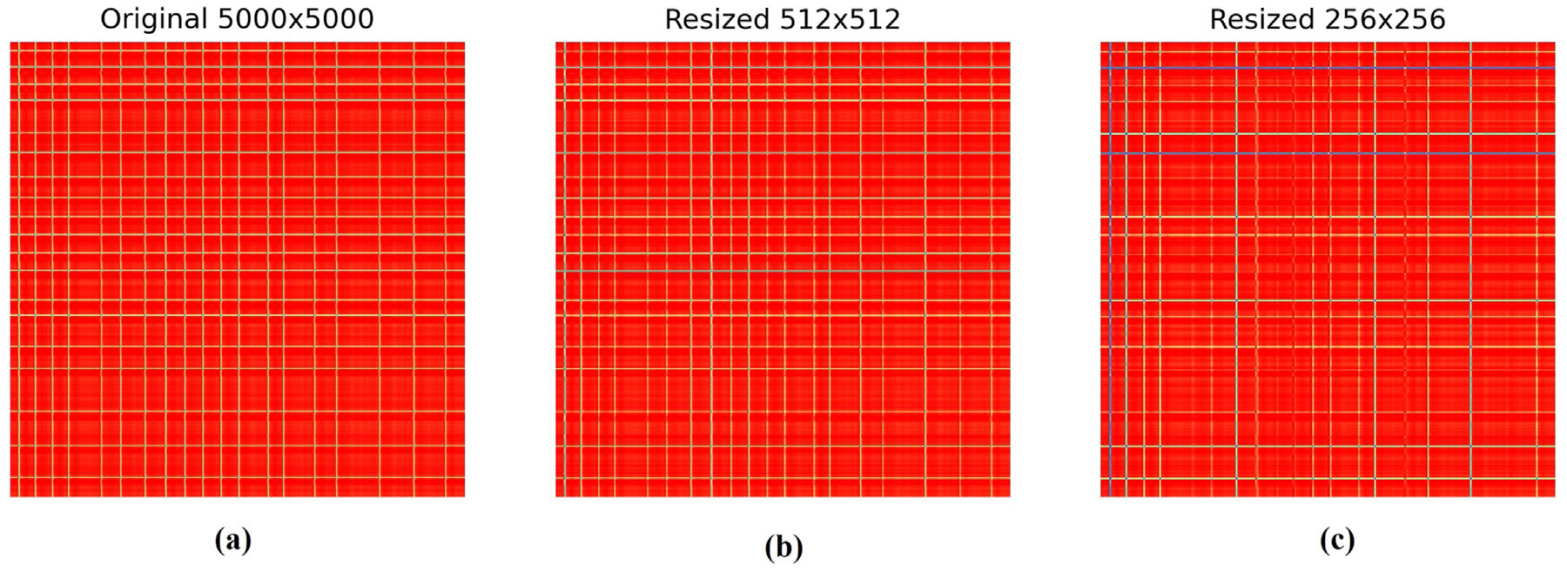
GAF Size Variations (a) shows the original GAF at a resolution of 5000 × 5000 pixels, (b) presents the GAF resized to 512 × 512 pixels, **(c)** illustrates the GAF further resized to 256 × 256 pixels.

Fig. 4. demonstrates the effect of resizing Gramian Angular Field (GAF) images on the representation of ECG data. The figure displays three GAF images at different resolutions:

• **Subfigure (a)** shows the original GAF at a resolution of 5000 × 5000 pixels, which preserves the highest level of detail and captures the full complexity of the ECG data.
• **Subfigure (b)** presents the GAF resized to 512 × 512 pixels, representing a substantial reduction in resolution. While the main patterns and overall structure of the data are retained, some fine details and nuances start to diminish.
• **Subfigure (c)** illustrates the GAF further resized to 256 × 256 pixels. At this resolution, noticeable information loss occurs, as finer structures and intricate patterns are significantly blurred or lost, potentially affecting the model's ability to accurately classify the ECG conditions.

As the resolution decreases from 5000 × 5000 to 256 × 256, a progressive loss of information can be observed, evidenced by the fading and simplification of the grid-like patterns in the GAF images. This information loss may impact the classification performance of models relying on these images, as lower resolutions may not capture the detailed temporal and spatial relationships inherent in the ECG signals. The figure emphasizes the importance of choosing an optimal GAF size that balances computational efficiency and the retention of critical information for accurate ECG classification.

Segmentation methods were also applied to the GAF images, segmenting the records by 1250 sampling points. This approach aimed to isolate relevant portions of the ECG signal, potentially improving the focus of the deep learning model on key features associated with each class.

**Fig. 5:**
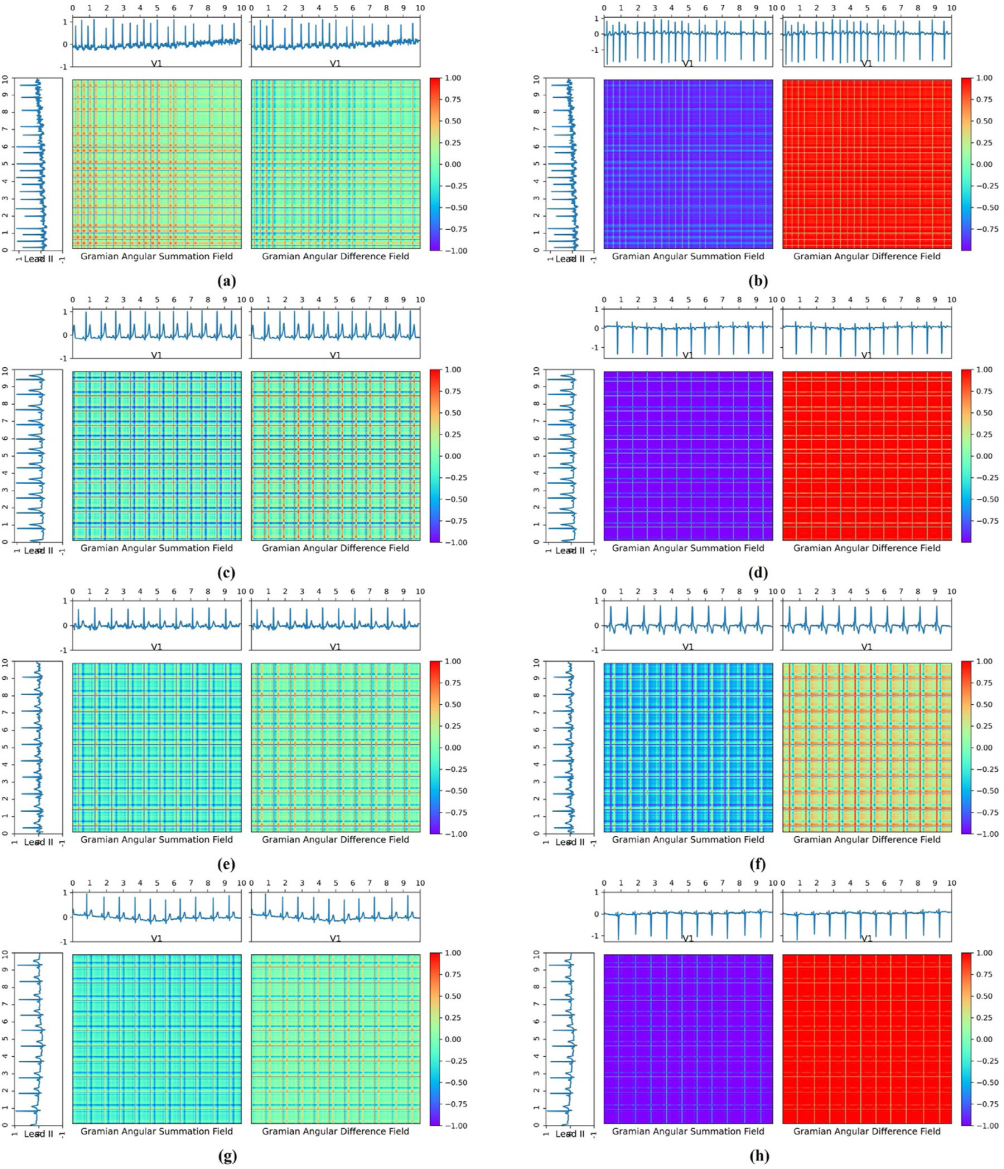
GAF transformation for Normal, Afib, RVH and LVH. Subfigures (a), (c), (e), and (g) represent GAF transformations of Lead I for Atrial Fibrillation (AFib), Left Ventricular Hypertrophy (LVH), Right Ventricular Hypertrophy (RVH), and Normal ECG, respectively. Correspondingly, subfigures (b), (d), (f), and (h) show the GAF transformations using both Lead I and V1 for the same conditions in the same order: AFib, LVH, RVH, and Normal.

Fig. 5. presents examples of GAF transformations applied to ECG signals for different cardiac conditions, using both single-lead and dual-lead configurations. Subfigures (a), (c), (e), and (g) represent GAF transformations of Lead I for Atrial Fibrillation (AFib), Left Ventricular Hypertrophy (LVH), Right Ventricular Hypertrophy (RVH), and Normal ECG, respectively. Correspondingly, subfigures (b), (d), (f), and (h) show the GAF transformations using both Lead I and V1 for the same conditions in the same order: AFib, LVH, RVH, and Normal.

### Classification model

A ConvNext architecture was employed to classify the GAF images into the four ECG categories. ConvNext is a modern CNN architecture designed to enhance traditional CNN capabilities by incorporating elements from vision transformers. It uses depthwise separable convolutions, layer normalization, and larger kernel sizes to capture broader contextual information. The ConvNext model was trained and evaluated on different GAF sizes and with/without segmentation to determine the optimal approach.

ConvNext is a modern convolutional neural network (CNN) architecture designed to enhance the capabilities of traditional CNNs by incorporating design elements from vision transformers. It evolves from the ResNet architecture, focusing on improving performance and scalability while retaining the efficiency of convolutional networks.

Key features of ConvNext include the use of depth wise separable convolutions to reduce computational cost, layer normalization for stable training, and larger kernel sizes for better context capture. It also employs residual connections and bottleneck layers to facilitate deeper network training, along with simplified activation functions like GELU for smoother gradient flow. By integrating attention-like mechanisms within the convolutional framework, ConvNext can effectively focus on important features, making it well-suited for complex image classification tasks.

In this study, ConvNext was used to classify ECG signals transformed into Gramian Angular Fields (GAFs), leveraging its ability to capture spatial hierarchies and subtle variations between different ECG leads.

## Method validation

### Classification performance

The classification performance was evaluated using accuracy, precision, recall, and F1-score metrics.

(3) $$Accuracy=\frac{True\;Positive+True\;Negative}{True\;positive+True\;Negative+False\;Positive+False\;Negative}$$

(3a) $$Precision=\frac{True\;Positive}{True\;positive+False\;Positive}$$

(4) $$Recall=\frac{True\;Positive}{True\;positive+False\;Negative}$$

(5) $$F1\;Score=\frac{2}{\frac{1}{Precision}+\frac{1}{Recall}}$$

The results are shown in Table 2. The table summarizes the classification performance of various models using different GAF image sizes and segmentation methods. The ConvNext model used, with GAF image sizes of 5000 × 5000, 512 × 512, and 256 × 256. Performance metrics include accuracy, precision, recall, and F1-score, measured across different experimental setups. The results indicate that the ConvNext model consistently outperformed other models across all GAF sizes, showing particularly high accuracy and F1-scores. The 512 × 512 GAF size with segmentation provided a balanced performance in terms of computational efficiency and accuracy. Larger GAF sizes (5000 × 5000) offered the highest performance but at the cost of increased computational resources, while the smallest size (256 × 256) showed a slight drop in performance, likely due to the loss of detailed features.

**Table 2:** F1-score results of test set data from PTB-XL.

Method	F1-score			
	A-fib	LVH	RVH	Normal
**5000×5000**	0.776	0.715	0.412	0.775
**512×512**	0.781	0.71	0.521	0.792
**256×256**	0.651	0.691	0.424	0.721
**2.5 Segmentation**	0.762	0.722	0.551	0.778

To evaluate the generalizability of the proposed method, we conducted validation experiments using the Chapman-Shaoxing database, a publicly available multi-lead ECG dataset [25]. The results, presented in Table 3, demonstrate the performance of the proposed classification model across different Gramian Angular Field (GAF) sizes and segmentation techniques. Compared to the original PTB-XL dataset results, the Chapman-Shaoxing validation results show consistent performance trends, confirming the robustness and generalizability of our approach across different ECG data sources. This validation further supports the efficacy of the GAF transformation and segmentation techniques in diverse clinical scenarios.

**Table 3:** F1-score results of test set data from Chapman dataset.

Method	F1-score			
	A-fib	LVH	RVH	Normal
**5000×5000**	0.751	0.704	0.398	0.768
**512×512**	0.774	0.706	0.504	0.789
**256×256**	0.612	0.681	0.414	0.732
**2.5 Segmentation**	0.759	0.713	0.546	0.756

### Impact of segmentation

Segmentation methods were observed to improve classification accuracy across all GAF sizes, with the most significant improvement seen in the 512 × 512 GAF images.

## Discussion

The findings of this study demonstrate that GAF transformation is an effective method for converting 1D ECG signals into a 2D format suitable for deep learning classification. The choice of GAF size significantly impacts both classification accuracy and computational requirements. Larger GAF sizes, such as 5000 × 5000, provide superior accuracy by preserving detailed temporal and spatial relationships within the ECG signals; however, they are computationally expensive and may not be practical for real-time or resource-constrained applications. Conversely, smaller GAF sizes, like 256 × 256, offer faster processing times and lower computational costs but may sacrifice some classification performance due to the loss of critical details. The intermediate size, 512 × 512, was found to strike a balance between computational efficiency and classification accuracy, especially when paired with segmentation techniques.

Segmentation methods were observed to enhance the performance of the classification model, likely by focusing the model on the most relevant parts of the ECG signal. By segmenting the data, the model could better capture the key features associated with specific cardiac conditions, such as AFib, LVH, and RVH. This finding suggests that further exploration of advanced segmentation techniques or the integration of feature selection methods could yield even better classification results.

For future work, we propose exploring multimodal studies that combine ECG data with other clinical data sources, such as echocardiography, patient demographics, or genetic information, to improve classification performance and robustness. Integrating multimodal data could provide a more comprehensive understanding of cardiovascular conditions and enhance the predictive power of the models. Additionally, further optimization of GAF transformation parameters and the exploration of other time-series encoding methods, such as Recurrence Plots or Markov Transition Fields, may yield improved representations of ECG data for classification purposes.

## Limitations

Despite the promising results, this study has several limitations. First, the analysis was limited to a specific set of ECG conditions and utilized the PTB-XL dataset, which may not fully represent the diversity of ECG patterns encountered in broader clinical settings. Additionally, the GAF transformation approach, while effective, relies on the transformation parameters that may not be optimized for all ECG signal characteristics. Another limitation is the focus on single and dual-lead configurations; while these setups offer valuable insights, the potential benefits of using additional leads or exploring different lead combinations were not exhaustively investigated. Furthermore, the study's models were trained on static ECG records, which do not account for temporal variations over longer periods, such as continuous monitoring data, which could impact model generalizability.

## Conclusion

This study demonstrates that transforming ECG signals into GAF images is a viable approach for classifying cardiac conditions such as AFib, LVH, RVH, and Normal ECG. The results highlight the trade-offs between GAF image size and classification performance, as well as the potential benefits of incorporating segmentation methods. The ConvNext model, when applied to GAF-transformed ECG data, showed strong performance, particularly when using a balanced GAF size with segmentation, underscoring the value of careful parameter selection in deep learning applications for ECG classification.

## Ethics statements

Human subjects, animal experiments, and social media platforms ethics are not relevant to our work.

## CRediT authorship contribution statement

**Gi-Won Yoon:** Conceptualization, Methodology, Software, Validation, Data curation, Writing – original draft, Visualization, Software, Validation, Writing – review & editing. **Segyeong Joo:** Conceptualization, Investigation, Supervision.

## Declaration of competing interest

The authors declare that they have no known competing financial interests or personal relationships that could have appeared to influence the work reported in this paper.

## Data Availability

Data will be made available on request.

## Appendix Supplementary materials

Image, application 1
