# Supplementary material for: Enhanced electrocardiogram classification using Gramian angular field transformation with multi-lead analysis and segmentation techniques
Source: MethodsX. 2025 Apr 8;14:103297. doi: 10.1016/j.mex.2025.103297 (PMC12033961; doi:10.1016/j.mex.2025.103297)
Supplement: Supplementary file 1 [file mmc1.docx]

**Supplementary material *and/or* additional information [OPTIONAL]**

Supplementary Table 1

5-Fold F1-Scores With 95% CI (PTB-XL Data)

| Method | Class | Fold1 | Fold2 | Fold3 | Fold4 | Fold5 | 95% CI |
| --- | --- | --- | --- | --- | --- | --- | --- |
| 5000x5000 | A-fib | 0.7639 | 0.7762 | 0.7715 | 0.7762 | 0.7922 | (0.7631, 0.7889) |
| 5000x5000 | LVH | 0.7128 | 0.7043 | 0.705 | 0.7181 | 0.7348 | (0.6995, 0.7305) |
| 5000x5000 | RVH | 0.416 | 0.414 | 0.406 | 0.3985 | 0.4255 | (0.3993, 0.4247) |
| 5000x5000 | Normal | 0.7696 | 0.7677 | 0.7694 | 0.7623 | 0.806 | (0.7532, 0.7968) |
| 512x512 | A-fib | 0.8008 | 0.7807 | 0.7741 | 0.7845 | 0.7649 | (0.7644, 0.7976) |
| 512x512 | LVH | 0.707 | 0.727 | 0.7097 | 0.7038 | 0.7025 | (0.6977, 0.7223) |
| 512x512 | RVH | 0.5174 | 0.5157 | 0.5333 | 0.5146 | 0.524 | (0.5113, 0.5307) |
| 512x512 | Normal | 0.7755 | 0.7883 | 0.7819 | 0.7932 | 0.8211 | (0.7702, 0.8138) |
| 256x256 | A-fib | 0.6656 | 0.6637 | 0.64 | 0.6283 | 0.6574 | (0.6309, 0.6711) |
| 256x256 | LVH | 0.6845 | 0.6696 | 0.7051 | 0.6765 | 0.7193 | (0.6653, 0.7167) |
| 256x256 | RVH | 0.4197 | 0.4235 | 0.4197 | 0.4258 | 0.4313 | (0.418, 0.43) |
| 256x256 | Normal | 0.7381 | 0.7295 | 0.7209 | 0.7338 | 0.6827 | (0.6933, 0.7487) |
| 2.5 Segmentation | A-fib | 0.7462 | 0.7636 | 0.7573 | 0.769 | 0.7739 | (0.7486, 0.7754) |
| 2.5 Segmentation | LVH | 0.7198 | 0.7395 | 0.7194 | 0.7229 | 0.7084 | (0.7081, 0.7359) |
| 2.5 Segmentation | RVH | 0.5435 | 0.5663 | 0.5693 | 0.5474 | 0.5285 | (0.53, 0.572) |
| 2.5 Segmentation | Normal | 0.7805 | 0.7694 | 0.7641 | 0.7707 | 0.8053 | (0.7577, 0.7983) |

Supplementary Table 2

5-Fold F1-Scores With 95% CI (Chapman Data)

| Method | Class | Fold1 | Fold2 | Fold3 | Fold4 | Fold5 | 95% CI |
| --- | --- | --- | --- | --- | --- | --- | --- |
| 5000x5000 | A-fib | 0.7397 | 0.7523 | 0.7489 | 0.7341 | 0.78 | (0.729, 0.773) |
| 5000x5000 | LVH | 0.6865 | 0.7068 | 0.7126 | 0.7124 | 0.7017 | (0.6906, 0.7174) |
| 5000x5000 | RVH | 0.412 | 0.4161 | 0.3897 | 0.3824 | 0.3898 | (0.3793, 0.4167) |
| 5000x5000 | Normal | 0.7672 | 0.753 | 0.7676 | 0.7687 | 0.7835 | (0.7546, 0.7814) |
| 512x512 | A-fib | 0.79 | 0.7788 | 0.7886 | 0.7902 | 0.7224 | (0.7377, 0.8103) |
| 512x512 | LVH | 0.7127 | 0.702 | 0.7146 | 0.7098 | 0.6909 | (0.6939, 0.7181) |
| 512x512 | RVH | 0.4986 | 0.5078 | 0.5141 | 0.5076 | 0.4919 | (0.4932, 0.5148) |
| 512x512 | Normal | 0.7916 | 0.7816 | 0.7884 | 0.7835 | 0.7999 | (0.78, 0.798) |
| 256x256 | A-fib | 0.6277 | 0.6079 | 0.6083 | 0.6127 | 0.6034 | (0.6004, 0.6236) |
| 256x256 | LVH | 0.6871 | 0.6889 | 0.6852 | 0.6726 | 0.6712 | (0.6705, 0.6915) |
| 256x256 | RVH | 0.4246 | 0.4044 | 0.4305 | 0.4025 | 0.408 | (0.3982, 0.4298) |
| 256x256 | Normal | 0.7489 | 0.7493 | 0.7366 | 0.7282 | 0.697 | (0.7053, 0.7587) |
| 2.5 Segmentation | A-fib | 0.7756 | 0.7512 | 0.7727 | 0.7688 | 0.7267 | (0.7337, 0.7843) |
| 2.5 Segmentation | LVH | 0.7225 | 0.7309 | 0.7191 | 0.7078 | 0.6847 | (0.6908, 0.7352) |
| 2.5 Segmentation | RVH | 0.5443 | 0.5475 | 0.5396 | 0.5355 | 0.5631 | (0.5328, 0.5592) |
| 2.5 Segmentation | Normal | 0.7618 | 0.7539 | 0.761 | 0.7587 | 0.7446 | (0.7472, 0.7648) |
